# C57BL/6J and C57BL/6NJ Mice Are Differentially Susceptible to Inflammation-Associated Disease Caused by Influenza A Virus

**DOI:** 10.3389/fmicb.2018.03307

**Published:** 2019-01-17

**Authors:** Amie J. Eisfeld, David J. Gasper, M. Suresh, Yoshihiro Kawaoka

**Affiliations:** ^1^Department of Pathobiological Sciences, University of Wisconsin–Madison, Madison, WI, United States; ^2^Division of Virology, Department of Microbiology and Immunology, The Institute of Medical Science, The University of Tokyo, Tokyo, Japan; ^3^International Research Center for Infectious Diseases, The Institute of Medical Science, The University of Tokyo, Tokyo, Japan

**Keywords:** influenza, pathogenicity, H1N1, H5N1, H7N9, inflammation, C57BL/6, C57BL/6N

## Abstract

Influenza viruses cause seasonal epidemics and sporadic pandemics, and are a major burden on human health. To develop better countermeasures and improve influenza disease outcomes, a clearer understanding of influenza pathogenesis is necessary. Host genetic factors have emerged as potential regulators of human influenza disease susceptibility, and in the mouse model, genetic background has been clearly linked to influenza pathogenicity. Here, we show that C57BL/6J mice are significantly more susceptible to disease caused by a 2009 pandemic H1N1 virus, an H7N9 virus, and a highly pathogenic H5N1 influenza virus compared to the closely related substrain, C57BL/6NJ. Mechanistically, influenza virus infection in C57BL/6J mice results in earlier presentation of edema, increased immune cell infiltration, higher levels of inflammatory cytokines, greater tissue damage, and delayed activation of regenerative processes in infected lung tissues compared to C57BL/6NJ mice. These differences are not dependent on virus replication levels. Six genes with known coding region differences between C57BL/6J and C57BL/6NJ strains exhibit increased transcript levels in influenza virus-infected mouse lungs, suggesting potential contributions to regulation of disease susceptibility. This work uncovers a previously unappreciated difference in disease susceptibility between the closely related C57BL/6J and C57BL/6NJ mice, which may be exploited in future studies to identify host factors and/or specific genetic elements that regulate host-dependent inflammatory mechanisms involved in influenza virus pathogenicity.

## Introduction

Influenza viruses cause seasonal epidemics in humans, resulting in an estimated 3 to 5 million cases of severe illness and 290,000 to 650,000 deaths worldwide annually (World Health Organization [WHO], 2018b), as well as pandemics that emerge at unpredictable intervals that may result in higher human morbidity and mortality. In addition, avian influenza viruses (including highly pathogenic H5N1 viruses and H7N9 viruses) cause sporadic infections in humans, typically associated with more severe disease and higher mortality rates compared to seasonal viruses (Food Agriculture Organization [FAO], 2018; World Health Organization [WHO], 2018a). Vaccines against seasonal influenza viruses are available, but the reported effectiveness is low {10–60% (Centers for Disease Control [CDC], 2018)}, and they are unlikely to protect against novel emerging pandemic strains. Antiviral drugs against influenza virus also are available, but emergence of resistance is a problem (Gubareva et al., 2017). Therefore, while influenza viruses pose multiple unique and important threats to human health, currently available countermeasures are insufficient to address these threats. A clearer understanding of the factors that increase susceptibility to severe influenza virus disease may enable more efficient distribution of available countermeasures to the most at-risk individuals in emergency pandemic scenarios; and furthermore, may provide insights that could permit the development of improved countermeasures in the future.

Common risk factors that are known to alter human influenza virus disease susceptibility include age, underlying comorbidities, and pregnancy; however, these risk factors do not explain all circumstances under which serious influenza-associated complications occur (Writing Committee of the WHO et al., 2010). Host genetic factors have emerged as potential regulators of human influenza disease susceptibility (Kenney et al., 2017), and may explain severe disease in otherwise apparently healthy individuals. In the mouse model, genetic background has been linked to differential virus replication (Srivastava et al., 2009; Boon et al., 2011; Otte et al., 2011; Pica et al., 2011; Trammell et al., 2012; Ferris et al., 2013), pro-inflammatory cytokine levels (Srivastava et al., 2009; Otte et al., 2011; Trammell et al., 2012; Boon et al., 2014), the severity and duration of pathological lesions (Srivastava et al., 2009; Ferris et al., 2013), and host transcriptional responses (Bottomly et al., 2012; Ferris et al., 2013; Wilk et al., 2015) in influenza virus-infected lungs; and to differences in susceptibility to influenza virus disease (Boon et al., 2009; Srivastava et al., 2009; Otte et al., 2011; Nedelko et al., 2012; Trammell et al., 2012). Moreover, quantitative trait loci mapping in recombinant inbred mouse strains has led to the identification of candidate genetic elements – and in a few cases, specific genetic variants – with (putative) roles in regulating influenza virus disease (Boon et al., 2011, 2014; Bottomly et al., 2012; Ferris et al., 2013). Thus, the mouse model has substantial potential for identifying genetic elements that are linked to influenza virus disease susceptibility.

The inbred C57BL/6J mouse is the reference strain for the *Mus musculus* genome sequence, is frequently used to generate knockout or knockin strains, and is a well-established model of influenza virus disease. C57BL/6J mice have been used extensively in mapping host genetic susceptibility to influenza viruses, typically as a founding component of the BXD genetic reference panel [which descends from C57BL/6J and DBA2/J mouse strains [Boon et al., 2009, 2014; Nedelko et al., 2012)], and more recently as one of the eight founding strains of the Collaborative Cross (Threadgill et al., 2011; Bottomly et al., 2012; Ferris et al., 2013). A unique but closely related substrain, C57BL/6NJ, is the strain used for all knockout mice generated under the International Knockout Mouse Consortium (Skarnes et al., 2011) and characterized by the International Mouse Phenotyping Consortium (Koscielny et al., 2014). C57BL/6J and C57BL/6NJ mice exhibit a variety of physiological and phenotypic differences; and small nucleotide polymorphisms, in-frame deletions, and structural variants affecting coding regions that differentiate C57BL/6J and C57BL/6NJ strains have been identified and validated (Simon et al., 2013). Based on this work, genetic coding variants that differ between the C57BL/6J and C57BL/6NJ strains have been attributed roles in regulation of hypertension (Leskov et al., 2017), inflammation (Aredo et al., 2015; Ulland et al., 2016), responses to cocaine and methamphetamine (Kumar et al., 2013), and binge eating (Kirkpatrick et al., 2017).

The use of C57BL/6NJ mice as an influenza virus disease model is rarely reported, and it is not clear whether C57BL/6J and C57BL/6NJ differ in their susceptibility to influenza virus disease. We reasoned that one or more of the genetic variants that differentiate C57BL/6J and C57BL/6NJ mice may regulate influenza virus disease susceptibility, and if so, such information could be not only essential for influenza researchers to design appropriate experiments with knockout mice, but also, an additional platform through which novel genetic regulators of influenza virus disease susceptibility might be identified. Therefore, the goal of this study was twofold: (i) to determine whether C57BL/6J and C57BL/6NJ differ in their susceptibility to influenza virus disease; and (ii) if differences in influenza virus disease susceptibility are apparent between strains, to determine the mechanism through which this difference occurs.

## Materials and Methods

### Ethics Statement

All animal experiments and procedures were approved by the University of Wisconsin (UW)-Madison School of Veterinary Medicine Animal Care and Use Committee, under relevant institutional and American Veterinary Association guidelines.

### Biosafety

All experiments using live H1N1 viruses were performed in biosafety level 2 (BSL-2) or animal enhanced biosafety level 2 (ABSL-2) containment laboratories at the UW-Madison. Experiments using live H5N1 or H7N9 viruses were performed in ABSL-3+ or BSL-3 agriculture (BSL-3Ag) containment laboratories, respectively, at the UW-Madison. UW-Madison BSL-2, ABSL-2, ABSL-3+, and BSL-3Ag laboratories are approved for use by the United States (US) Centers for Disease Control and Prevention (CDC) and the US Department of Agriculture.

### Cells

Madin-Darby canine kidney (MDCK) cells were propagated in minimum essential medium containing 5% newborn calf serum, and 293T human embryonic kidney cells were propagated in Dulbecco’s modified Eagle’s medium containing 10% fetal bovine serum. All cells were maintained at 37°C in an atmosphere of 5% CO_2_. Cell stocks are periodically restarted from early passage aliquots and routinely monitored for mycoplasma contamination.

### Viruses

The A/California/04/09 H1N1 virus (CA04) was provided by the United States Centers for Disease Control and Prevention (CDC). The A/Vietnam/1203/2004 (H5N1) virus (VN1203), originally provided by the United States CDC, was rescued by reverse genetics as described previously (Neumann et al., 1999; Watanabe et al., 2008). The A/Anhui/1/2013 (H7N9) virus (AH1), originally provided by Yuelong Shu (director of the World Health Organization [WHO] Collaborating Center for Reference and Research on Influenza, director of the Chinese National Influenza Center, and deputy director of the National Institute for Viral Disease Control and Prevention China CDC, Beijing, People’s Republic of China), was rescued by reverse genetics, as described previously (Neumann et al., 1999; Yamayoshi et al., 2014). Stock viruses were generated by passaging an aliquot of the original virus (CA04) or supernatants derived from reverse genetics transfection experiments (VN1203 and AH1) one time in MDCK cells containing 0.6% bovine serum albumin (BSA) fraction V (Sigma-Aldrich) and 1 μg/ml tosyl phenylalanyl chloromethyl ketone (TPCK)-trypsin, as previously described (Eisfeld et al., 2014). Stock virus titers were quantified by plaque assay in MDCK cells using standard methods.

### Mouse Infections

Eight- to ten-week-old C57BL/6J or C57BL/6NJ mice (The Jackson Laboratory) were anesthetized by intraperitoneal (i.p.) injection of ketamine and dexmedetomidine (45–75 mg/kg ketamine + 0.25–1 mg/kg dexmedetomidine) and were intranasally inoculated with 50 μl of phosphate-buffered saline (PBS; “mock”) or PBS containing viruses at the dosages indicated in the text, figures and figure legends. Following inoculation, dexmedetomidine was reversed by i.p. injection of atipamezole (0.1–1 mg/kg). Subsequent to infection, individual body weights and survival were monitored for up to 14 days. Median lethal dose (LD_50_) experiments were terminated at 12 days post-infection to minimize personnel time spent in BSL-3 containment. Mice were humanely euthanized when exhibiting severe clinical symptoms, at the end of the observation period, or at designated time points for tissue collection. LD_50_ values were calculated according to the method of Reed and Muench (1938).

### Lung Sample Collection

For lung tissue collection experiments, lungs were dissected and preserved for different analyses as follows: the right superior lobe was collected for virus titration and frozen at −80°C in the absence of buffer; the right inferior lobe was collected for cytokine quantification and frozen at −80°C in the absence of buffer; the right middle and post-caval lobes were directly submerged in Invitrogen^TM^ RNA*later*^TM^ Stabilization Solution, then placed at 4°C overnight, followed by freezing at −80°C; and the left lung tissues were preserved by immersion in 10% phosphate-buffered formalin.

### Virus Titration in Lung Tissues

Lung tissue portions (the right superior lobes) were thawed, weighed, and then homogenized in 1 ml of PBS containing a penicillin/streptomycin mixture by using a TissueLyser II (Qiagen) at 30-Hz oscillation frequency for 3 min. Homogenates were centrifuged (10,000 × *g* for 10 min) to remove debris, and virus titers in cleared homogenate supernatants were determined by plaque assays in MDCK cells using standard methods. Virus titers were normalized to plaque forming units (pfu) per gram (g) of lung tissue.

### Cytokine Assays

Lung tissue portions (right inferior lobes) were thawed and homogenized in 500 μl of PBS containing 0.5% BSA and a protease inhibitor cocktail (Complete^TM^, Mini, EDTA-free, Sigma-Aldrich) by using a TissueLyser II at 30-Hz oscillation frequency for 3 min. Homogenates were centrifuged (10,000 × *g* for 10 min) to remove debris, and cleared homogenate supernatants were used for cytokine quantification with the Bio-Plex Pro^TM^ Mouse Cytokine 23-plex Assay (Bio-Rad; assayed cytokines include CCL11, CSF3, CSF2, IFNG, IL1A, IL1B, IL2, IL3, IL4, IL5, IL6, IL9, IL10, IL12A, IL12B, IL13, IL17A, CXCL1, CCL2, CCL3, CCL4, CCL5, and TNF) and the Bio-Plex^TM^ 200 system according to the manufacturer’s instructions. Cleared homogenate supernatants also were used for type I interferon quantification with the VeriKine Mouse Interferon Alpha ELISA kit (PBL Assay Science) and the VeriKine Mouse Interferon Beta ELISA Kit (PBL Assay Science) according to the manufacturer’s instructions. ELISAs were evaluated with a Tecan Infinite M1000 plate reader.

### Histopathology

Following fixation, lung tissues (left lobes) were paraffin-embedded and processed for routine histopathology. Five micron-thick sections were stained with hematoxylin and eosin and examined by light microscopy. Blinded scoring of histologic lesions was performed by using an ordinal scale from 0 to 5, with 0 indicating no lesion, and numbers 1–5 indicating the presence of a lesion and its severity and extent: 1, minimal; 2, mild; 3, moderate; 4, marked; and 5, severe. Separate scoring was performed for large airways (bronchi and bronchioles) and terminal airways and alveoli (for a list of scored lesions, see Supplementary Table S1).

### RNA Isolation and Quantitative Reverse Transcriptase Polymerase Chain Reaction (qPCR)

For RNA isolation, lung tissues (right middle and post-caval lobes submerged in Invitrogen^TM^ RNA*later*^TM^) were thawed, transferred to 1 ml Invitrogen^TM^ TRIzol^TM^, and homogenized by using a TissueLyser II at 30-Hz oscillation frequency for 3 min. Following 10 min incubation at room temperature, homogenates were centrifuged (10,000 × *g* for 10 min) to remove debris, and cleared homogenate supernatants were used for RNA extraction with the miRNeasy^TM^ mini kit (Qiagen) according to the manufacturer’s instructions. First-strand cDNA synthesis was performed using 1 μg of total RNA and the QuantiTect^TM^ reverse transcription kit (Qiagen), according to the manufacturer’s instructions. The qPCR assay was performed with one fifth of each cDNA reaction, 500 nM of each PrimeTime^TM^ gene-specific primer set (Integrated DNA Technologies), and the PowerUp^TM^ SYBR^TM^ green master mix (Applied Biosystems), according to the manufacturer’s instructions. qPCR assays were performed with the QuantStudio 6 Flex Real-Time PCR System (Applied Biosystems). Relative RNA quantities were determined by using the comparative threshold cycle method, with the mouse Tbp gene serving as the endogenous reference and mock-infected samples serving as the calibrators. All primer sequences are provided in Table 1.

**Table 1:** qPCR primer sequences.

Gene symbol	Assay part number	Primer sequences	
Chl1	Mm.PT.58.11844622	5′-CATGCGTATGGTACCGATCAC-3′
		5′-CACTGCGAGTACTTTGCTTCT-3′
Plk1	Mm.PT.58.12563595	5′-GCTGTAGCAAGTCACTAAGGT-3′
		5′-GCAGCAGGAAACCTCTCAA-3′
Cyfip2	Mm.PT.58.30699273	5′-ACACTCTCCATCCTTCCCAT-3′
		5′-CCACGCGCTACAATTATACCA-3′
Adamts3	Mm.PT.58.33524777	5′-CCCACACTTATCCTCAACCTT-3′
		5′-GCTCTTACAAAGATCCATACAGCA-3′
Nlrp12	Mm.PT.58.28813850	5′-ACATGCTTTGGAGGTGAGTC-3′
		5′-GGACCTGAGCTTCAATGACTT-3′
Pdzk1	Mm.PT.58.17572080	5′ACAAGGAGGAACATGACGG-3′
		5′-GAATGGAGAAAATGTAGAGAACGC-3′
Tbp	Mm.PT.39a.22214839	5′-CCAGAACTGAAAATCAACGCAG-3′
		5′-TGTATCTACCGTGAATCTTGGC-3′

### Statistical Analyses

Individual mouse body weight ratios (that is, weight at a given time point/weight immediately prior to infection) and group lung histopathology scores were compared by using the non-parametric Mann–Whitney test with exact *p*-value calculations. Survival curves were compared by using the log-rank Mantel–Cox test. To enable statistical comparisons, lung cytokines that were not detected in any condition were assigned a value equal to one half the lower limit of quantification for the respective assay. Lung virus titers (PFU/g), lung cytokines (PFU/ml), and lung transcript levels (determined by qPCR) were log_10_ transformed and compared by using the two-tailed Student’s *t*-tests without assuming consistent standard deviation. For lung cytokines and lung transcript levels, *t*-tests were followed by a Holm–Sidak post-test. Correlations between cytokines and body weights were determined by using the Pearson correlation coefficient. All statistical analyses were performed with GraphPad Prism 7 software.

### Microarray Data

Published microarray datasets GSE33263 (IM001) and GSE37569 (CA04M001) were previously analyzed (McDermott et al., 2011; Tchitchek et al., 2013) and are available in the NCBI Gene Expression Omnibus (GEO) database^1^ under the aforementioned accession numbers.

## Results

### C57BL/6J and C57BL/6NJ Mice Are Differentially Susceptible to Pandemic H1N1 Influenza A Virus Disease

**FIGURE 1:**
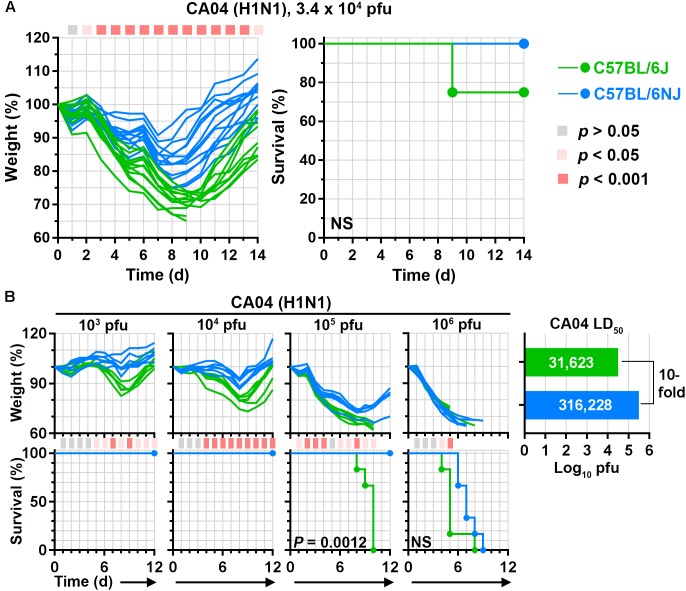
C57BL/6J and C57BL/6NJ mice exhibit differential susceptibility to pandemic H1N1 influenza A virus disease. **(A)** Groups of C57BL/6J or C57BL/6NJ mice (12–13 mice for each strain) were intranasally infected with 3.4 × 10^4^ plaque forming units (pfu) of influenza A/California/04/2009 (H1N1; ‘CA04’), and body weights and survival were monitored for 14 days. Body weight ratio profiles (that is, weight at a given time point/weight immediately prior to infection) of individual mice are shown, and the colored square corresponding to each time point (at the top of the graph) indicates whether a statistically significant difference was observed between C57BL/6J and C57BL/6NJ mice. Survival curves are not significantly different. NS, not significant; d, day. **(B)** Groups of six C57BL/6J or C57BL/6NJ mice were intranasally infected with serial dilutions of CA04 and body weight ratios and survival were monitored for 12 days. Body weight ratios and significance are shown as in **(A)**, except colored squares indicating statistical significance are shown at the bottom of the weight loss panels. For survival curves that significantly differ between C57BL/6J and C57BL/6NJ mice, *p*-values are given on the survival curve panel. Median lethal dose (LD_50_) values were calculated for each mouse strain by using the Reed and Muench method (Reed and Muench, 1938), and are shown in the plot at the right.

We have used C57BL/6J mice extensively to evaluate disease after infection with different influenza A viruses (Hatta et al., 2010; McDermott et al., 2011; Shinya et al., 2012; Tchitchek et al., 2013; Josset et al., 2014; Shoemaker et al., 2015; Chasman et al., 2016). However, it is unclear whether C57BL/6J mice and the closely related C57BL/6NJ strain exhibit similar susceptibility to influenza A viruses. To compare influenza A virus-associated disease in C57BL/6J and C57BL/6NJ mice, we infected each mouse strain with a human 2009 H1N1 virus (A/California/04/2009; ‘CA04’) at a dosage previously determined to be equivalent to ∼1 median lethal dose (LD_50_) in C57BL/6J mice and monitored individual body weights and survival for 14 days. While both C57BL/6J and C57BL/6NJ mice were susceptible to CA04-induced disease, C57BL/6NJ weight loss was significantly less than that of C57BL/6J mice from days 3–14 post-infection, and C57BL/6NJ mice recovered starting body weights sooner than C57BL/6J mice (Figure 1A, left). In addition, while 23% of the C57BL/6J mice succumbed to the infection, all of the C57BL/6NJ mice survived (Figure 1A, right). To formally quantify the differences in CA04-mediated disease, we infected C57BL/6J and C57BL/6NJ mice with serial dilutions of CA04 to determine the LD_50_ for each mouse strain. C57BL/6NJ mice exhibited significantly less weight loss [see the 10^3^, 10^4^, and 10^5^ plaque-forming unit (pfu) dosages], significantly increased survival (see the 10^5^ pfu dosage), and a 10-fold higher LD_50_ compared to C57BL/6J mice (Figure 1B). Together, these observations indicate that C57BL/6J mice are more susceptible to pandemic H1N1 disease compared to C57BL/6NJ mice.

### C57BL/6J and C57BL/6NJ Mice Are Differentially Susceptible to H7N9 and Highly Pathogenic H5N1 Influenza A Virus Disease

**FIGURE 2:**
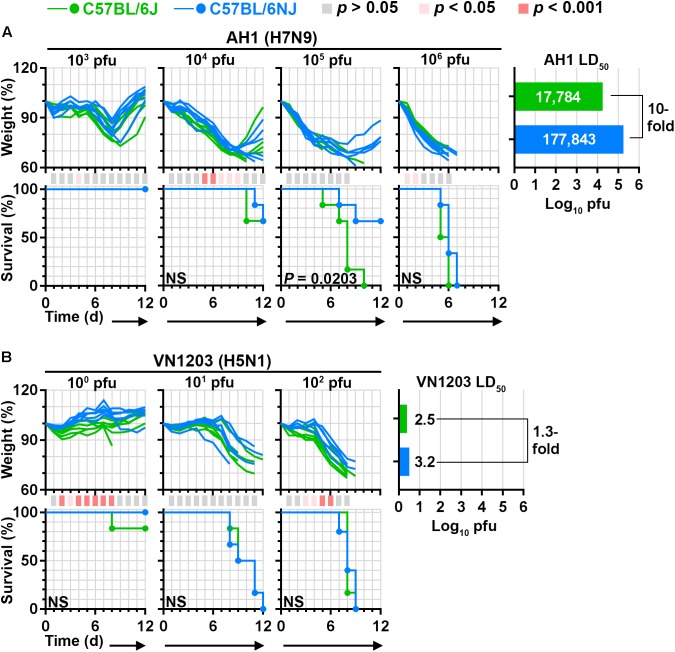
C57BL/6J and C57BL/6NJ mice exhibit differential susceptibility to H7N9 and highly pathogenic H5N1 influenza virus disease. Groups of five or six C57BL/6J or C57BL/6NJ mice were intranasally infected with serial dilutions of **(A)** influenza A/Anhui/1/2013 (H7N9; ‘AH1’), or **(B)** influenza A/Vietnam/1203/2004 (H5N1; ‘VN1203’), and body weight ratios and survival were monitored for 12 days. Body weight ratios and significance are shown as in Figure 1B. For survival curves that significantly differ between C57BL/6J and C57BL/6NJ mice, *p*-values are given on the survival curve panel. Median lethal doses were calculated for each virus in each mouse strain by using the Reed and Muench method (Reed and Muench, 1938), and are shown at the right of each panel. NS, not significant; d, day.

To determine whether differences in C57BL/6J and C57BL/6NJ susceptibility to influenza A virus disease extend to other virus strains, we carried out LD_50_ experiments with a human H7N9 virus (A/Anhui/1/2013; ‘AH1’) and a human highly pathogenic H5N1 virus (A/Vietnam/1203/2004; ‘VN1203’). Overall, AH1 caused greater weight loss than CA04 in mice infected with the same dosages (Figure 2A, compare to Figure 1B), consistent with previous observations (Morrison et al., 2014). Similar to CA04 infection, AH1 infection induced significantly less weight loss (see the 10^4^ pfu dosage), significantly greater survival (see the 10^5^ pfu dosage), and a 10-fold increase in LD_50_ in C57BL/6NJ mice relative to C57BL/6J mice (Figure 2A). As expected from our previous study (Tchitchek et al., 2013), VN1203 was highly pathogenic in C57BL/6J mice; and while no differences in survival or LD_50_ were observed between C57BL/6J and C57BL/6NJ mice, C57BL/6NJ mice exhibited significantly less weight loss at the highest evaluated infection dosage (Figure 2B). Altogether, these observations indicate that C57BL/6J mice are more susceptible to disease caused by multiple influenza A viruses compared to C57BL/6NJ mice, irrespective of established differences in virus pathogenicity.

### Influenza A Virus Replicates Similarly in Lungs of C57BL/6J and C57BL/6NJ Mice, but Causes More Severe Respiratory Disease in C57BL/6J Mice

**FIGURE 3:**
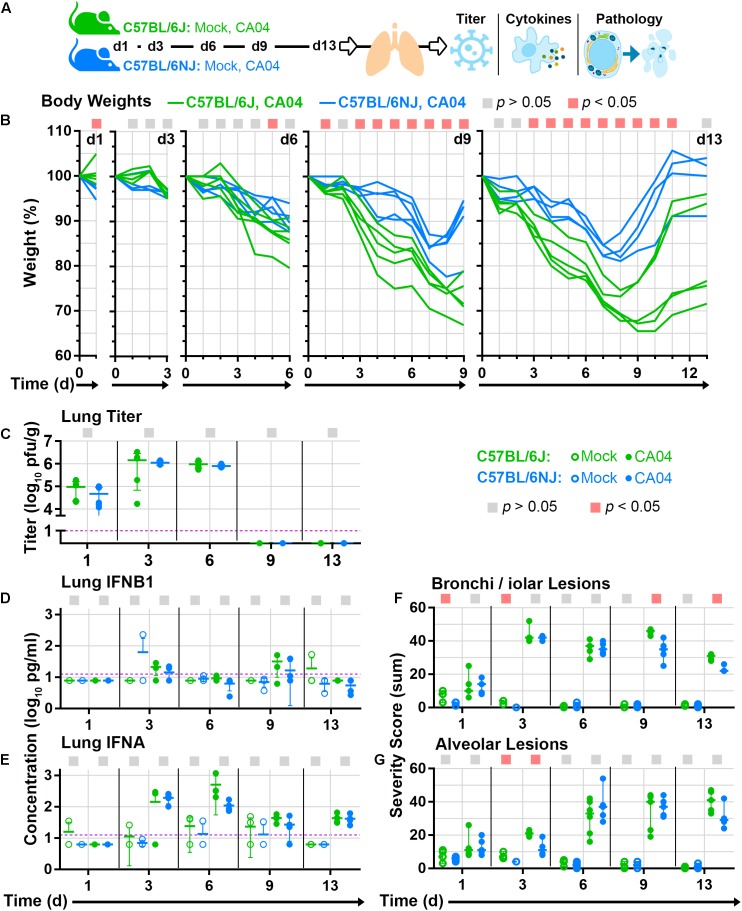
Lung tissue analyses. Groups C57BL/6J or C57BL/6NJ mice were mock-infected (with PBS) or infected with a sub-lethal dosage (3.4 × 10^4^ pfu) of CA04, euthanized at five time points [day 1 (d1), d3, d6, d9, and d13; four to six mice were euthanized per group at each time point], and lung tissues were collected and divided for various analyses. **(A)** Overview of mouse lung tissue collection time points and subsequent analyses. **(B)** Individual mouse body weight ratio profiles are shown for CA04-infected C57BL/6J and C57BL/6NJ mice in each time point group. **(C)** Individual lung titers are shown for each CA04-infected mouse at each time point. Concentrations of **(D)** IFNB1 or **(E)** IFNA in lung homogenates are shown for individual mock-infected and CA04-infected mice at each time point. Individual histopathology sum severity scores of **(F)** bronchi/iolar lesions and **(G)** alveolar lesions are shown for mock-infected and CA04-infected mice at each time point (see Supplementary Table S1 for all histopathology scoring data). In **(A–G)**, the colored squares at the top of each graph indicate statistically significant differences between C57BL/6J and C57BL/6NJ for a given comparison. In **(C–E)**, group means are shown by horizontal bars, error bars indicate standard deviation, and the limit of detection is represented by a dashed purple line. In **(F,G)** group medians are shown by horizontal bars and error bars indicate the 95% confidence interval. d, day; pg, picograms; g, gram; ml, milliliter.

Differences in susceptibility to influenza virus disease may be caused by factors that regulate virus replication levels, or alternatively, factors that regulate host responses that contribute to disease pathogenesis. To distinguish between these possibilities, we next mock-infected (with phosphate-buffered saline, PBS) or infected groups of mice with a sub-lethal dosage of CA04 (3.4 × 10^4^ pfu), and collected lung tissues at 5 time points (1, 3, 6, 9, and 13 days post-infection) for virus titration, cytokine quantification, and histopathology analysis (Figure 3A). Despite clear differences in C57BL/6J and C57BL/6NJ body weights after CA04 infection (Figure 3B) – similar to those observed in our previous experiments (Figure 1) – lung virus titers exhibited the same overall kinetics, peak levels, and timing of resolution in both mouse strains (Figure 3C). Lung concentrations of type I interferons, IFNB1 (Figure 3D) or IFNA (Figure 3E), were also similar between C57BL/6J and C57BL/6NJ mice, with IFNA exhibiting higher expression than IFNB1, and IFNA reaching peak levels concurrent with peak virus titers in both mouse strains. Although no differences in lung virus replication or type I interferon expression were observed between C57BL/6J and C57BL/6NJ mice, the sum severity scores of histological changes in bronchioles (Figure 3F) and alveoli (Figure 3G) suggested more severe disease in C57BL/6J mice (see Supplementary Table S1 for all histopathology scoring data). These observations suggest that the genetic factors controlling differential susceptibility of C57BL/6J and C57BL/6NJ mice to influenza virus disease do not regulate virus replication levels or type I interferon induction in the lung, but rather, host responses that contribute to influenza virus disease pathogenesis.

### C57BL/6J Mice Exhibit Increased Lung Edema at Early Times After Influenza A Virus Infection

**FIGURE 4:**
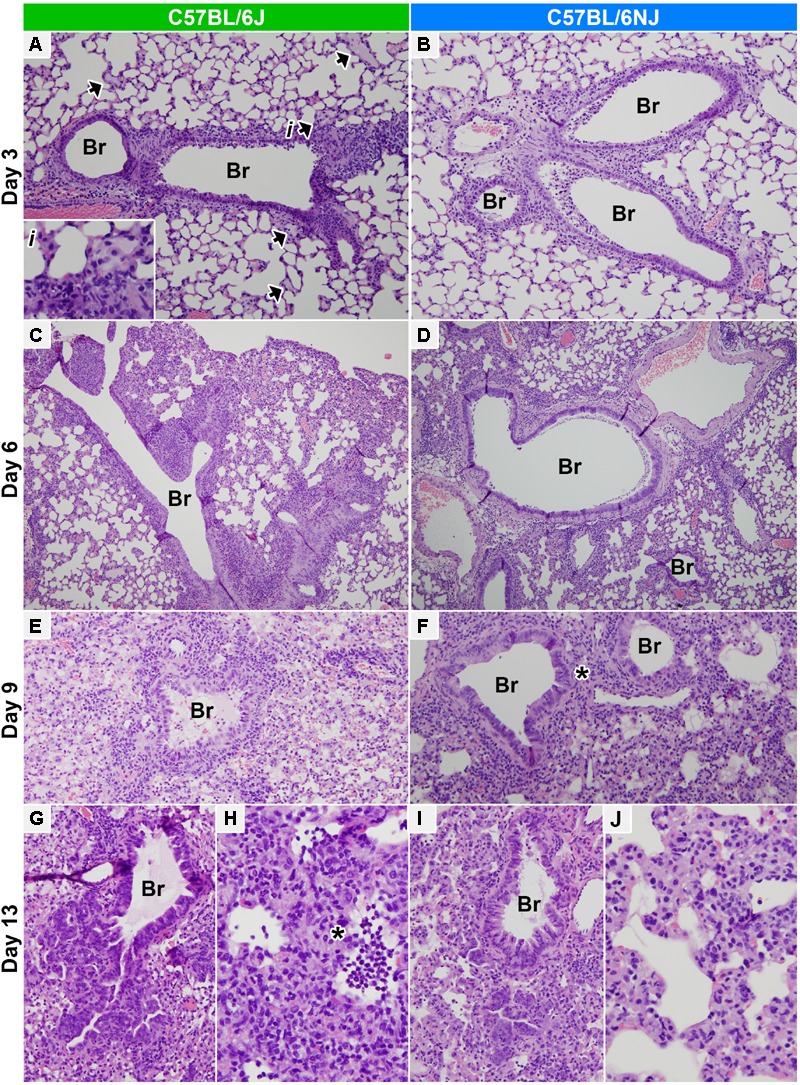
Lung histopathology. Hematoxylin and eosin stained lung tissue sections from CA04-infected C57BL/6J **(A,C,E,G,H)** or C57BL/6NJ **(B,D,F,I,J)** mice. **(A,B)** Medium bronchioles and adjacent alveolar parenchyma on day 3 post-infection (100× magnification). Necrotizing bronchiolitis is prominent in both C57BL/6J and C57BL/6NJ mice, but acute alveolar damage and edema in the parenchyma abutting affected airways are more pronounced in C57BL/6J mice [see the arrowheads and inset (i) in **A**]. **(C,D)** Large bronchioles and adjacent alveolar parenchyma on day 6 post-infection (40× magnification). Lungs of C57BL/6J and C57BL/6NJ mice are similar, with necrotizing bronchiolitis slightly less severe than day 3, and locally severe to regionally extensive peribronchiolar and subpleural alveolar damage and pneumonia. **(E,F)** Small bronchioles and adjacent alveolar parenchyma on day 9 post-infection (100× magnification, with 3.5-fold enlargement). Interstitial pneumonia and diffuse alveolar damage are widespread both C57BL/6J and C57BL/6NJ mice, with slightly more extensive alveolar disease in C57BL/6J mice. Regions of prominent segmental bronchiolar epithelial hyperplasia occur in both strains, but are more prominent in C57BL/6NJ mice [see asterisk (^∗^) in **F**]. **(G,I)** Terminal bronchioles and adjacent alveolar parenchyma on day 13 post-infection (100× magnification, with threefold enlargement). Many small and terminal airways exhibit prominent, deeply basophilic hyperplastic columnar epithelium which extends into adjacent alveoli. **(H,J)** Alveolar parenchyma on day 13 post-infection (200× magnification, with threefold enlargement). Alveolar necrosis is more prominent in C57BL/6J mice [see asterisk (^∗^) in **H**]. See Supplementary Table S1 for all histopathology scoring data. Br, bronchi/ioles.

To pinpoint specific differences in influenza disease pathogenesis between C57BL/6J and C57BL/6NJ strains, we next examined histopathology assessments in more detail. On day 1 post-infection, mock- and CA04-infected mice of both strains exhibited minimal to mild multifocal acute swelling and necrosis of bronchiolar respiratory epithelial cells; and mild to moderate regional changes in the alveolar walls due to expansion of alveolar walls by edema, increased circulating leukocytes, and increased numbers of foamy macrophages within the interstitium of the alveolar walls (Supplementary Table S1). Bronchiolar lesions were slightly more severe in mock-infected C57BL/6J mice compared to mock-infected C57BL/6NJ mice, but no differences were observed between strains in CA04-infected mice (Figure 3F). On day 3 post-infection, mock-infected C57BL/6J mice had a slightly higher frequency of individual cell necrosis in the bronchiolar respiratory epithelium, and slightly more prominent expansion of the alveolar walls with edema (Figures 3F,G and Supplementary Table S1). In CA04-infected mice of both strains, airways exhibited moderate multifocal necrotizing bronchiolitis primarily affecting the medium- to large-caliber airways (Figures 4A,B). In addition, minimal multifocal acute alveolar damage was observed in parenchyma abutting affected airways, with mild alveolar edema and increased numbers of luminal granulocytes in the most severely affected areas in C57BL/6J mice (Figure 4A, arrows and inset; and Supplementary Table S1). These observations suggest that: (i) C57BL/6J mice may be predisposed to a more aggressive response in the lung, given that histological lesions in the bronchioles were significantly greater in mock-infected C57BL/6J mice compared to mock-infected C57BL/6NJ mice at early time points after infection; and (ii) influenza virus infection in C57BL/6J mice may lead to more rapid and/or greater disruption of epithelial and/or endothelial barrier integrity, potentially as a result of increased granulocyte extravasation, leading to significantly increased peribronchiolar and alveolar edema at early times after infection compared to C57BL/6NJ mice.

### C57BL/6J Mice Exhibit Greater Immune Cell Infiltrates, Increased Lung Damage, and Delayed Regenerative Responses at Late Times After Influenza A Virus Infection

On day 6 post-infection, background lesions in mock-infected mice of both strains were mostly resolved (Supplementary Table S1), and lesions in CA04-infected mice were highly similar (Figures 3F,G and Supplementary Table S1). In CA04-infected mice, necrotizing bronchiolitis was slightly less severe but more wide spread than at day 3, with some bronchiolar epithelium exhibiting moderate segmental hyperplasia; moderately to densely cellular infiltrates of neutrophils, eosinophils, and macrophages encircled the most severely affected airways and adjacent vessels (bronchovascular cuffing); and locally severe to regionally extensive peribronchiolar and subpleural alveolar damage and pneumonia was observed in some mice (Figures 4C,D). On day 9 post-infection, CA04-infected C57BL/6J mice were more severely affected than CA04-infected C57BL/6NJ mice, with higher levels of necrotizing bronchiolitis in small airways (Figures 4E,F and Supplementary Table S1), greater multifocal lymphohistiocytic and neutrophilic peribronchiolar cuffing (Supplementary Table S1), and more intraepithelial granulocytes in bronchioles (Supplementary Table S1). Both strains exhibited regions of segmental bronchiolar epithelial hyperplasia, which was more prominent in C57BL/6NJ mice (Figure 4F, asterisk), suggesting greater activation of regenerative processes at this time point. In both mouse strains, interstitial pneumonia and diffuse alveolar damage were more widespread than on day 6 – including loss of alveolar architectural definition, with either extensive luminal effusion and dense cellular infiltrates (foamy macrophages, lymphocytes and neutrophils) (Figures 4E,F), or extensive necrotic cellular debris with small numbers of neutrophils and lymphocytes (data not shown). On day 13 post-infection, bronchiolitis was largely resolved in both strains and many small and terminal airways exhibited prominent, respiratory epithelial hyperplasia that extended into adjacent alveoli (Figures 4G,I). In contrast, there was a marked increase in the extent of the alveolar disease in both mouse strains, which was more severe in C57BL/6J mice, including variably sized regions of alveolar necrosis with effusion; accumulation of luminal necrotic debris, fibrin, neutrophils and macrophages; and hemorrhage (Figures 4H,J and Supplementary Table S1). Altogether, these observations indicate greater immune cell infiltrates, increased lung tissue damage, and delayed regenerative responses in C57BL/6J mice at late times after influenza A virus infection.

### Influenza A Virus Infection Induces Higher Expression of Inflammatory Cytokines in C57BL/6J Lungs

**FIGURE 5:**
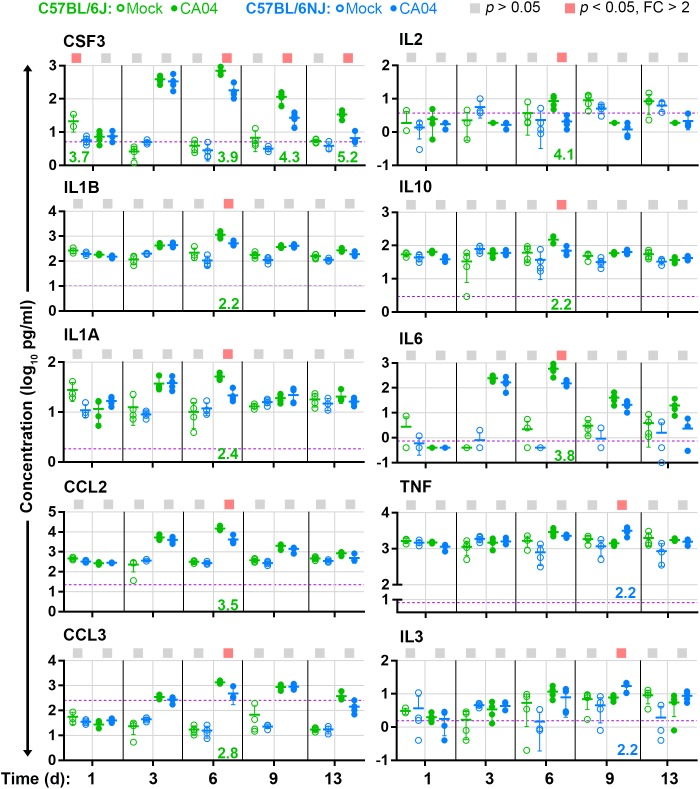
Lung cytokine levels. For cytokines that are differentially expressed (fold change [FC] > 2, Holm–Sidak-adjusted *p* < 0.05) in concentration between CA04-infected C57BL/6J and C57BL/6NJ mice in at least one time point, the concentrations [picograms (pg) per ml] in lung homogenates are shown for individual mock-infected and CA04-infected mice for all time points. Group means are shown by horizontal bars, error bars indicate standard deviation, and the limit of detection is represented by a dashed purple line. Colored squares at the top of each graph indicate statistically significant differences between C57BL/6J and C57BL/6NJ for a given comparison. Fold-changes associated with statistically significant comparisons are indicated at the bottom of each graph (green text indicates higher expression in C57BL/6J mice, and blue text indicates higher expression in C57BL/6NJ mice). See Supplementary Table S2 for all cytokine data.

High inflammatory cytokines levels are associated with increased immune cell infiltrates and greater tissue damage in influenza A virus-infected lung tissues (Liu et al., 2016). Since C57BL/6J mice exhibit greater immune cell infiltrates and increased lung tissue damage after influenza A virus infection compared to C57BL/6NJ mice, we reasoned that inflammatory cytokines may be induced at higher levels in C57BL/6J mice. To test this hypothesis, we measured and compared levels of 23 cytokines in lung tissue homogenates of C57BL/6J and C57BL/6NJ mice (Supplementary Table S2). Most cytokines were induced with similar kinetics in C57BL/6J and C57BL/6NJ mice after CA04 infection (Supplementary Table S2); however, a subset exhibited higher expression in C57BL/6J mice at one or more time points (Figure 5). CSF3, which promotes the production, differentiation, and function of granulocytes, was significantly higher in C57BL/6J mice at days 6, 9, and 13 days post-infection (Figure 5), consistent with higher levels of peribronchiolar cuffing and bronchiolar intraepithelial granulocytes in C57BL/6J mice on days 9 and 13 post-infection (Supplementary Table S1). At day 6 post-infection, C57BL/6J mice also exhibited higher levels of IL1A, IL1B, CCL2, CCL3, IL2, IL10, and IL6 (Figure 5). In contrast, two cytokines – TNF and IL3 – exhibited significantly higher levels in C57BL/6NJ mice on day 9 post-infection (Figure 5). These observations show that, overall, inflammatory cytokine expression is higher in C57BL/6J mice, and that higher levels of TNF and IL3 may be associated with resolution of inflammation in C57BL/6NJ mice.

### Lung Inflammatory Cytokines Are Correlated to Body Weight in Influenza A Virus-Infected C57BL/6J Mice

**FIGURE 6:**
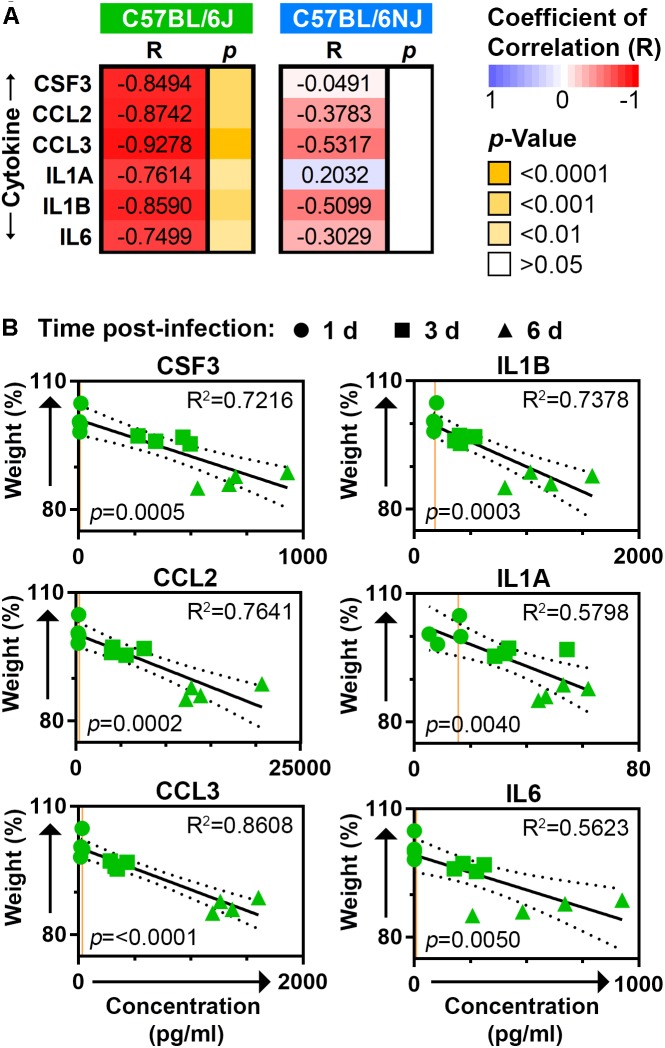
Correlation between mouse body weights and lung cytokines. **(A)** The heat map shows Pearson coefficients of correlation (*R*) and corresponding *p*-values for body weights and lung cytokine measurements obtained from the CA04-infected C57BL/6J and C57BL/6NJ mice on days 1, 3, and 6 post-infection. **(B)** Scatter plots show matching body weight and lung cytokine measurements for C57BL/6J mice. In each plot, the time post-infection for each set of measurements is indicated by circles (day 1), squares (day 3), or triangles (day 6); the best fit linear regression is shown by a solid line with 95% confidence intervals indicated by dotted lines; coefficients of determination (*R*^2^) are given at the upper right; and *p*-values are given at the lower left. Orange lines represent the average expression in mock-infected samples for the given cytokine.

To more clearly establish whether excessive inflammatory cytokine expression may be driving influenza disease severity, we determined the Pearson coefficient of correlation (R) for body weights and lung cytokine measurements obtained from the CA04-infected mice on days 1, 3, and 6 post-infection. All six inflammatory cytokines that exhibited significantly higher levels in C57BL/6J mice at one or more of these time points (CSF3, CCL2, CCL3, IL1A, IL1B, and IL6; see Figure 5) were strongly and significantly negatively correlated to matched body weights in C57BL/6J mice (*R* ≤ −0.7499, *p* ≤ 0.01), but not C57BL/6NJ mice (Figure 6A). Linear regression analysis further revealed strong coefficients of determination (*R*^2^) for CSF3, CCL2, CCL3, and IL1B (*R*^2^ ≥ 0.7216) in C57BL/6J mice (Figure 6B), indicating that inflammatory cytokine lung concentrations may be sufficient to explain most body weight variation in C57BL/6J mice at days 1, 3, and 6 post-infection. Together, these observations support the notion that an excessive immune response propels greater disease severity in C57BL/6J mice, while a more constrained immune response has little impact on disease severity in C57BL/6NJ mice.

### A Subset of Host Genes That Vary Between C57BL/6J and C57BL/6NJ Mice Are Differentially Expressed in Mouse Lung After Influenza Infection

**FIGURE 7:**
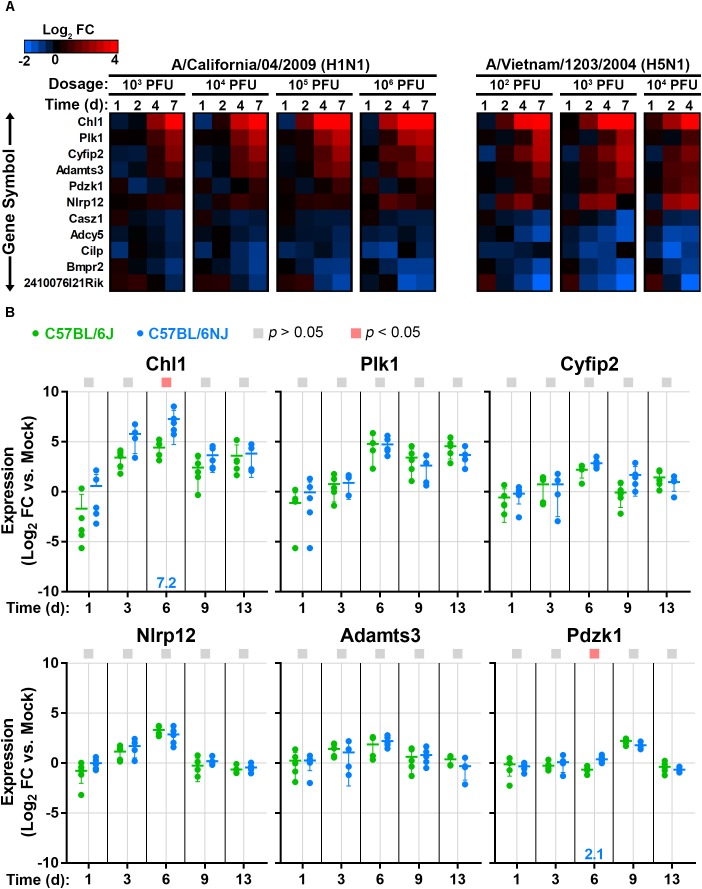
Lung expression of host genes that vary between C57BL/6J and C57BL/6NJ mice after influenza infection. **(A)** The heat map shows host genes that are differentially expressed (fold change > 1.5, false discovery rate-adjusted *p*-value < 0.05) in at least one dosage and time point for both CA04 and VN1203, as determined from previously published microarray analysis of C57BL/6J mouse lung tissue (GSE33263 and GSE37569) (McDermott et al., 2011; Tchitchek et al., 2013). **(B)** For the six host genes that were up-regulated in C57BL/6J mouse lungs in **(A)**, relative RNA quantities were determined in C57BL/6J and C57BL/6NJ by using qPCR with the comparative threshold cycle method, with the mouse Tbp gene serving as the endogenous reference and mock-infected samples serving as the calibrators. Group means are shown by horizontal bars, and error bars indicate standard deviation. Statistical significance and the associated fold-changes are reported as described for Figure 5.

Previously, 51 coding region small nucleotide polymorphisms, in-frame deletions, or structural variants that differentiate C57BL/6J and C57BL/6NJ mice were identified and validated (Simon et al., 2013). To determine whether influenza virus induces significant changes in the expression of these genes in mouse lung, which could imply a role in the response to influenza virus infection, we queried previously published microarray datasets from CA04- and VN1203-infected C57BL/6J mice (McDermott et al., 2011; Tchitchek et al., 2013). We identified 11 genes that were significantly altered after infection with both viruses (Figure 7A), and among these genes, 6 were up-regulated after infection (Chl1, Nlrp12, Plk1, Cyfip2, Adamts3, and Pdzk1). Using quantitative real-time reverse transcriptase PCR of RNA extracted from mock-infected and CA04-infected lung tissues (from the experiment described in Figure 3), we evaluated the expression levels of these six genes in C57BL/6J and C57BL/6NJ mice (Figure 7B). All six genes were up-regulated in both mouse strains, with kinetics that closely aligned to that of the previous microarray experiment in C57BL/6J mice. Notably, Chl1 was significantly more highly expressed in C57BL/6NJ mice, consistent with a potential role in reduced inflammation, decreased immune cell infiltration, and/or decreased tissue damage after influenza virus infection.

## Discussion

In this study, we compared influenza virus disease susceptibility in two closely related but unique mouse strains – C57BL/6J and C57BL/6NJ – and demonstrated that C57BL/6J mice are significantly more susceptible than C57BL/6NJ mice to influenza viruses that are associated with variable levels of disease in humans. We further demonstrated that while influenza virus replication in the lung was similar between C57BL/6J and C57BL/6NJ mice, the more susceptible C57BL/6J mice exhibited earlier presentation of pulmonary edema, increased immune cell infiltration, higher levels of inflammatory cytokines, greater tissue damage, and delayed activation of regenerative processes in infected lung tissues compared to C57BL/6NJ mice. Therefore, we have identified a mouse genetic model system in which influenza disease susceptibility is a consequence of an excessive immune response uncoupled from the level of influenza virus replication. This system may be useful for identifying host factors and/or specific genetic elements that regulate host-dependent mechanisms involved in influenza virus pathogenicity.

Although our data clearly indicate increased severity of microscopic lesions and higher cytokine levels in influenza virus-infected C57BL/6J mice compared to C57BL/6NJ mice, the precise mechanisms that regulate the differences in susceptibility remain to be determined. Most of the cytokines that exhibit significantly increased expression in C57BL/6J mice are predominantly expressed by activated macrophages (CSF3, IL1A, IL1B, CCL3, and IL10), or are pleiotropically expressed by multiple cell types, including monocytes and macrophages (CCL2 and IL6), suggesting that macrophage activation may be more robust in C57BL/6J mouse lungs. Macrophages in C57BL/6J mice may possess an inherent capacity for higher cytokine production, or alternatively, a reduced capacity to regulate cytokine levels. In turn, higher expression of pro-inflammatory cytokines could mediate increased tissue damage through increased immune cell recruitment and/or activation, disruption of barrier integrity, or induction of epithelial cell death. However, it is also possible that higher levels of intrinsic apoptosis or necrosis in virus-infected or bystander epithelial cells could provide increased stimuli for macrophage activation, leading to higher cytokine expression and further tissue damage. Interestingly, TNF (which also is predominantly expressed by macrophages) exhibited higher expression in C57BL/6NJ mice at late time points, coinciding with earlier recovery in these animals. Previous studies have demonstrated that TNF is required to limit pulmonary immunopathology and tissue remodeling in influenza virus infections in mice (Damjanovic et al., 2011; DeBerge et al., 2014), which is consistent with reduced pathology and subdued tissue remodeling in C57BL/6NJ mice at later time points. Overall, our observations suggest that differences in macrophage activation profiles could regulate the outcomes of influenza virus infections in C57BL/6J and C57BL/6NJ mice, and this needs to be further examined.

The host genetic element(s) responsible for the difference in influenza virus pathogenicity between C57BL/6J and C57BL/6NJ mice currently are not known. In previous studies, the roles of dozens of host factors in regulating influenza virus disease susceptibility have been assessed in knockout mouse models (mostly in the C57BL/6J background), but to our knowledge, most host factors that possess validated genetic differences in coding regions between C57BL/6J and C57BL/6NJ mice have not been evaluated. In the lungs of both C57BL/6J and C57BL/6NJ mice, we observed up-regulation of six genes with established coding region differences between these mouse strains (Chl1, Nlrp12, Plk1, Cyfip2, Adamts3, and Pdzk1), and we suggest that any of these genes potentially could control influenza disease susceptibility. Of particular interest, the Nlrp12 gene – which encodes a small nucleotide polymorphism (SNP) resulting in a lysine (in C57BL/6J) or an arginine (in C57BL/6NJ) at amino acid 1034 (Simon et al., 2013), within the most C-terminal leucine-rich domain – has been shown to positively or negatively regulate inflammatory responses (including IL1B expression) and/or immune cell infiltration depending on the context (Allen et al., 2012; Vladimer et al., 2012; Zaki et al., 2014; Ulland et al., 2016; Chen et al., 2017; Silveira et al., 2017); and a recent study demonstrated that Nlrp12 knockout mice are protected from lethal influenza infection, most likely due to reduced levels of pulmonary neutrophil infiltration (Hornick et al., 2018). We suggest that the Nlrp12 SNP that differentiates C57BL/6J and C57BL/6NJ mice could modulate pro-inflammatory cytokine expression in macrophages responding to influenza virus infection; or alternatively, could alter neutrophil recruitment into influenza virus-infected lungs. Also of high interest is the Chl1 gene, which encodes a long interspersed nuclear element transposon in one of its introns in C57BL/6J mice (Simon et al., 2013), and is more highly expressed in lungs of C57BL/6NJ mice after influenza virus infection. Chl1 is a cell adhesion molecule expressed on myeloid, lymphoid, and epithelial cells (Felding-Habermann et al., 1997), and has been shown to bind to CD24, which is highly expressed on neutrophils (Elghetany and Patel, 2002; Hernandez-Campo et al., 2007) and, upon cross-ligation, accelerates neutrophil death (Parlato et al., 2014). We suggest that increased Chl1 expression in influenza virus-infected C57BL/6NJ lungs could lead to timely neutrophil death and resolution of inflammation, whereas lower expression in infected C57BL/6J lungs could lead to prolonged neutrophil survival, resulting in higher levels of neutrophil-mediated tissue damage and inflammation. The identification of host genetic elements that regulate differential influenza virus pathogenicity in C57BL/6J and C57BL/6NJ mice is currently under investigation.

Recently, others have shown that there are substantial differences in the microbiota of mice purchased from different commercial vendors or repositories, and that that these differences can affect experimental outcomes (Franklin and Ericsson, 2017). Since the mice used in this study were obtained from the same source, we consider it unlikely that differences in microbiota that are dependent on commercial source or husbandry practices affected the results of the current study. However, it is possible that genetic differences between C57BL/6J and C57BL/6NJ mice could affect the microbiota, which in turn, could affect the susceptibility of these mice to influenza virus disease. This possibility needs to be explored in future experiments.

In summary, this work establishes a clear difference in influenza virus pathogenicity mediated by host inflammatory processes in the closely related C57BL/6J and C57BL/6NJ mouse strains. This information will be important to aid influenza researchers in designing appropriate experiments in knockout mouse models, and further, provides a new platform through which novel genetic regulators of influenza virus disease susceptibility might be identified in further studies.

## Author Contributions

AE and YK conceptualized the study. AE designed the methodology, performed the experiments, and analyzed the data. DG performed histopathology analyses. The original manuscript draft and visualizations were prepared by AE and reviewed and edited by YK, DG, and MS. YK and MS supervised the project. Funding was acquired by YK, MS, and DG.

## Conflict of Interest Statement

The authors declare that the research was conducted in the absence of any commercial or financial relationships that could be construed as a potential conflict of interest.

## Footnotes

1 https://www.ncbi.nlm.nih.gov/geo/
